# Supplementary material for: Polyketide synthase gene clusters in the ericoid mycorrhizal fungus Oidiodendron maius: genome-wide prediction, expression profiling, and functional characterization of knockout mutants
Source: Front Plant Sci. 2026 Sep 16;17:1924430. doi: 10.3389/fpls.2026.1924430 (PMC13623903; doi:10.3389/fpls.2026.1924430)
Supplement: Supplementary file 1 [file SupplementaryFile1.zip › Supplementary Materials/Supplementary Folder 1.zip!/Oidma1_AssemblyScaffolds_Repeatmasked/knownclusterblast/cluster40/jgi.p_Oidma1_133494_mibig_hits.html]

| MIBiG Protein | Description | MIBiG Cluster | MiBiG Product | % ID | % Coverage | BLAST Score | E-value |
| --- | --- | --- | --- | --- | --- | --- | --- |
| BAE56588.1 |  | BGC0001123 | Aspirochlorine\_biosynthetic\_gene\_cluster | 53 | 99.6233521657 | 561 | 8.7e-160 |
| EAQ86388.1 | hypothetical\_protein | BGC0001405 | Chaetoviridin\_/\_chaetomugilin\_biosynthetic\_gene\_cluster | 50 | 100.753295669 | 485 | 1e-136 |
| AGO59046.1 | PtaM | BGC0000121 | Pestheic\_acid\_biosynthetic\_gene\_cluster | 46 | 100.0 | 466 | 5e-131 |
| ALT31850.1 | ArmH3 | BGC0001401\_c3 | Melleolides\_biosynthetic\_gene\_cluster | 46 | 100.188323917 | 462 | 5.5e-130 |
| AEM76785.1 | putative\_halogenase | BGC0001401 | Melleolides\_biosynthetic\_gene\_cluster | 46 | 100.376647834 | 461 | 1.2e-129 |
| ADI24948.1 | GsfI | BGC0000070 | Griseofulvin\_biosynthetic\_gene\_cluster | 46 | 99.8116760829 | 453 | 3.3e-127 |
| AEM76786.1 | putative\_halogenase | BGC0001401\_c2 | Melleolides\_biosynthetic\_gene\_cluster | 45 | 100.564971751 | 444 | 1.6e-124 |
| ALT31851.1 | ArmH4 | BGC0001401\_c4 | Melleolides\_biosynthetic\_gene\_cluster | 46 | 101.506591337 | 436 | 5.5e-122 |
| ACD39771.1 | non-heme\_halogenase | BGC0000134 | Radicicol\_biosynthetic\_gene\_cluster | 43 | 100.753295669 | 419 | 5.4e-117 |
| ALT31852.1 | ArmH5 | BGC0001401\_c5 | Melleolides\_biosynthetic\_gene\_cluster | 42 | 99.8116760829 | 413 | 2.9e-115 |
| ABM21576.1 | CrpH | BGC0000975 | Cryptophycin\_biosynthetic\_gene\_cluster | 38 | 74.1996233522 | 264 | 2.2e-70 |
| AMK51270.1 | Hex13 | BGC0001376 | Hexaricin\_biosynthetic\_gene\_cluster | 37 | 79.2843691149 | 256 | 7.8e-68 |
| ADE22292.1 | putative\_non-heme\_halogenase | BGC0000279 | Xantholipin\_biosynthetic\_gene\_cluster | 35 | 77.5894538606 | 255 | 1.3e-67 |
| CAM34371.1 | putative\_non-heme\_halogenase | BGC0000242 | Lysolipin\_biosynthetic\_gene\_cluster | 36 | 79.2843691149 | 248 | 2.1e-65 |
| CAJ46693.1 | tryptophan\_halogenase | BGC0000969 | Chondramide\_biosynthetic\_gene\_cluster | 33 | 83.615819209 | 229 | 7.9e-60 |
| ACU71639.1 | tryptophan\_halogenase | BGC0001154 | Cacibiocin\_biosynthetic\_gene\_cluster | 31 | 72.6930320151 | 205 | 2.1e-52 |
| AEU17894.1 | putative\_halogenase | BGC0001072 | Simocyclinone\_biosynthetic\_gene\_cluster | 32 | 73.8229755179 | 203 | 4.6e-52 |
| WP\_052390547.1 | halogenase | BGC0001308 | Simocyclinone\_D9\_/\_simocyclinone\_D10\_/\_simocyclinone\_D11 | 33 | 73.0696798493 | 202 | 1.3e-51 |
| ADU85999.1 | putative\_tryptophan\_halogenase | BGC0000165 | Tiacumicin\_B\_biosynthetic\_gene\_cluster | 31 | 80.2259887006 | 188 | 2.6e-47 |
| CAE53362.1 | halogenase | BGC0000440 | Teicoplanin\_biosynthetic\_gene\_cluster | 30 | 75.8945386064 | 170 | 7.4e-42 |
| CAG15020.1 | halogenase | BGC0000441 | Teicoplanin\_biosynthetic\_gene\_cluster | 30 | 75.8945386064 | 170 | 7.4e-42 |
| AAK81830.1 | non-heme\_halogenase | BGC0000326 | Complestatin\_biosynthetic\_gene\_cluster | 32 | 76.647834275 | 169 | 9.7e-42 |
| AAM80532.1 | StaI | BGC0000290 | A47934\_biosynthetic\_gene\_cluster | 27 | 83.9924670433 | 167 | 3.7e-41 |
| AAN65237.1 | putative\_halogenase | BGC0000832 | Clorobiocin\_biosynthetic\_gene\_cluster | 31 | 65.1600753296 | 162 | 1.2e-39 |
| AEI58871.1 | halogenase | BGC0000455 | Vancomycin\_biosynthetic\_gene\_cluster | 30 | 75.7062146893 | 162 | 1.5e-39 |
| CAD91205.1 | halogenase | BGC0000289 | A40926\_biosynthetic\_gene\_cluster | 31 | 74.5762711864 | 160 | 4.5e-39 |
| AGS77325.1 | Halogenase | BGC0001178 | UK-68,597\_biosynthetic\_gene\_cluster | 28 | 75.8945386064 | 159 | 1e-38 |
| CAA76550.1 | halogenase | BGC0000311 | Balhimycin\_biosynthetic\_gene\_cluster | 30 | 76.0828625235 | 158 | 2.2e-38 |
| CAA11780.1 | PCZA361.26 | BGC0000322\_c2 | Chloroeremomycin\_biosynthetic\_gene\_cluster | 29 | 76.2711864407 | 156 | 1.1e-37 |
| ctg1\_orf12 |  | BGC0001366 | BE-24566B\_/\_zunyimycin\_A\_biosynthetic\_gene\_cluster | 31 | 66.1016949153 | 155 | 2.5e-37 |
| AFV52210.1 | aryl-ACP\_halogenase | BGC0000081 | Kedarcidin\_biosynthetic\_gene\_cluster | 28 | 74.1996233522 | 153 | 7.2e-37 |
| AAL06656.1 | alkylhalidase | BGC0000965 | C-1027\_biosynthetic\_gene\_cluster | 29 | 73.4463276836 | 151 | 2.1e-36 |
| ALU98528.1 | tryptophan\_halogenase | BGC0001397 | Lidamycin\_biosynthetic\_gene\_cluster | 29 | 72.3163841808 | 151 | 3.6e-36 |
| ABS50488.1 | NapH2 | BGC0000652 | Napyradiomycin\_biosynthetic\_gene\_cluster | 29 | 69.868173258 | 150 | 6.1e-36 |
| ABS50460.1 | NapH2 | BGC0001079 | Napyradiomycin\_biosynthetic\_gene\_cluster | 29 | 69.868173258 | 150 | 6.1e-36 |
| CAQ43074.1 | halogenase | BGC0000970 | Chondrochloren\_biosynthetic\_gene\_cluster | 29 | 74.1996233522 | 148 | 3e-35 |
| ABD65950.1 | halogenase | BGC0000341 | Enduracidin\_biosynthetic\_gene\_cluster | 28 | 72.8813559322 | 147 | 5.1e-35 |
| ALD82533.1 | halogenase | BGC0001212 | Nannocystin\_biosynthetic\_gene\_cluster | 29 | 72.1280602637 | 146 | 8.8e-35 |
| ABY66006.1 | halogenase | BGC0001008 | Maduropeptin\_biosynthetic\_gene\_cluster | 29 | 73.8229755179 | 143 | 7.4e-34 |
| AAM54090.1 | halogenase | BGC0000020 | Ansamitocin\_biosynthetic\_gene\_cluster | 30 | 72.8813559322 | 141 | 2.8e-33 |
| ADM46362.1 | halogenase | BGC0000106 | Naphthomycin\_biosynthetic\_gene\_cluster | 29 | 66.2900188324 | 138 | 2.4e-32 |
| ACE80255.1 | HrmQ | BGC0000374 | Hormaomycin\_biosynthetic\_gene\_cluster | 27 | 80.790960452 | 127 | 5.5e-29 |
| AAM80530.1 | StaK | BGC0000290 | A47934\_biosynthetic\_gene\_cluster | 27 | 69.1148775895 | 126 | 7.2e-29 |
| AAM70353.1 | CalO3 | BGC0000033 | Calicheamicin\_biosynthetic\_gene\_cluster | 27 | 66.4783427495 | 120 | 6.7e-27 |
| AHA34035.1 | Bmp2 | BGC0000890 | Bromophenols\_/\_bromopyrroles\_biosynthetic\_gene\_cluster | 26 | 66.2900188324 | 106 | 7.7e-23 |
| ESP90845.1 | Dehydrogenases\_(flavoprotein) | BGC0000891 | Bromophenols\_/\_bromopyrroles\_biosynthetic\_gene\_cluster | 25 | 66.1016949153 | 104 | 5e-22 |
| AGC24269.1 | PrlO | BGC0001038 | Pyralomicin\_biosynthetic\_gene\_cluster | 23 | 73.4463276836 | 57 | 4.1e-08 |
